# Supplementary material for: Integrated Bioinformatics and Machine Learning for Ascertainment and Validation of Biomarkers for Screening Breast Disease
Source: Genes (Basel). 2025 Nov 18;16(11):1389. doi: 10.3390/genes16111389 (PMC12652564; doi:10.3390/genes16111389)
Supplement: Supplementary file 1 [file genes-16-01389-s001.zip › Supplementary Table S2.pdf]

Supplementary Table S2

Full form	Abbreviation
Gene Expression Omnibus	GEO
Weighted Gene Co-expression Network Analysis	WGCNA
Differentially Expressed Genes	DEGs
Benign Breast Disease	BBD
Breast Cancer	BC
Machine Learning	ML
Linear Models for Microarray Data	LIMMA
Least Absolute Shrinkage and Selection Operator Regression	LASSO Regression
Random Forest	RF
Support Vector Machine Recursive Feature Elimination	SVM-RFE
Receiver Operating Characteristic	ROC
Immune Cell Infiltration	IC Infiltration
Arrestin Domain Containing 1	ARRDC1
ATPase Sarcoplasmic/Endoplasmic Reticulum Ca2+ Transporting 2	ATP2A2
BC Detection Demonstration Project	BCDDP
Proliferative Breast Disease	PD
Topological Overlap Matrix	TOM
Gene Set Enrichment Analysis	GSEA
Principal Component Analysis;	PCA
Volcano Plot	VP
Biological Process	BP
Cellular Component	CC
Breast Cancer Stem Cells	BCSCs
Gene Ontology	GO
Chromosomal Instability	CIN
Kyoto Encyclopedia of Genes and Genomes	KEGG
